# Cultivar-Dependent Variation in Silicon Accumulation and Phytolith Formation Revealed by Comparative Proteomic Analysis in Rice

**DOI:** 10.3390/ijms27219917

**Published:** 2026-11-05

**Authors:** Ratchapon Prakongkaew, Wasinee Pongprayoon

**Affiliations:** Department of Biology, Faculty of Science, Burapha University, Chon Buri 20131, Thailand

**Keywords:** *Oryza sativa* L., silicon, phytolith, proteomics, LC-MS/MS, silicon transporter proteins, protein–protein interaction, climate resilience, rice

## Abstract

Silicon is a key determinant of growth, productivity, and stress adaptation in rice (*Oryza sativa* L.). Silicon accumulation and its deposition as phytoliths contribute to plant performance and environmental resilience. However, although the physiological functions of silicon and the mechanisms governing its uptake and transport have been extensively studied, the proteomic basis underlying cultivar-dependent variation in silicon accumulation and phytolith formation remains poorly understood. In this study, silicon concentration, phytolith content, and proteomic profiles were compared in roots and shoots of three rice cultivars, Chai Nat 1 (CNT), Khao Dawk Mali 105 (KDML105), and RD51, at the flowering stage. RD51 exhibited the highest silicon concentration and phytolith accumulation in shoots, whereas CNT accumulated the highest silicon concentration in roots. Proteomic analysis identified 4086 and 4115 protein groups in roots and shoots, respectively, revealing cultivar-specific differences in protein abundance. Principal component analysis and differential abundance analysis demonstrated the greatest proteomic divergence between CNT and RD51, particularly in shoots. Functional enrichment analysis indicated that KDML105 was enriched in transport- and sulfur metabolism-related processes, whereas RD51 showed enrichment of mitochondrial respiration, energy production, and associated metabolic pathways. Protein–protein interaction (PPI) network analysis further revealed functional associations between silicon transporter proteins and proteins involved in transport, signaling, and respiratory metabolism. Collectively, these findings provide novel insights into the proteomic mechanisms underlying silicon accumulation and phytolith formation in rice. The superior silicon accumulation and phytolith production observed in RD51 highlight its potential as a valuable genetic resource for enhancing phytolith production, improving silicon-use efficiency, and strengthening climate resilience in rice.

## 1. Introduction

Rice (*Oryza sativa* L.) is one of the most important cereal crops worldwide and serves as the primary food source for more than half of the global population. Improving rice productivity and resilience to environmental stresses is therefore essential for sustainable food security [1,2]. Silicon (Si) is the second most abundant element in the Earth’s crust and is accumulated by many plant species. Rice is recognized as one of the highest silicon-accumulating crops and can contain up to 10% Si on a shoot dry-weight basis [3,4]. Silicon is absorbed in the form of monosilicic acid [Si(OH)_4_] and subsequently transported throughout the plant, where it is deposited as amorphous silica within tissues. Silicon accumulation has been associated with enhanced plant growth, improved photosynthetic efficiency, increased resistance to diseases and insect pests, reduced lodging, and greater tolerance to abiotic stresses including drought, salinity, and flooding [5]. In rice, silicon uptake and distribution are mediated by specialized transporter proteins. Low Silicon 1 (Lsi1), a member of the nodulin 26-like intrinsic protein (NIP) family, acts as an influx transporter responsible for silicon uptake into root cells, whereas Low Silicon 2 (Lsi2) functions as an efflux transporter that mediates silicon movement toward the stele for xylem loading [6,7]. In aerial tissues, Low Silicon 6 (Lsi6) facilitates silicon unloading from vascular tissues, while Low Silicon 3 (Lsi3) contributes to silicon redistribution to leaves and reproductive organs [8,9]. Following transport, silicon is deposited as hydrated silica bodies known as phytoliths, which contribute to cell-wall reinforcement, structural support, and improved tolerance to environmental stresses [10,11]. The capacity for silicon uptake, translocation, and phytolith formation varies considerably among rice cultivars because of differences in genetic background and physiological characteristics [12,13]. Although the physiological roles of silicon and silicon transporters have been extensively investigated, the molecular mechanisms associated with cultivar-dependent variation in silicon accumulation and phytolith formation remain poorly understood. In particular, information regarding cultivar-specific proteins associated with silicon utilization at the flowering stage is still limited. Recent advances in liquid chromatography–tandem mass spectrometry (LC-MS/MS)-based proteomics have enabled comprehensive characterization of proteins involved in nutrient transport, metabolism, development, and stress responses [14,15]. Comparative proteomic analysis provides a powerful approach for identifying proteins and biological processes associated with specific physiological traits and environmental adaptations. Therefore, integrating physiological measurements with proteomic analysis may provide new insights into the molecular mechanisms governing silicon accumulation and phytolith formation in rice. Chai Nat 1 (CNT), Khao Dawk Mali 105 (KDML105), and RD51 are rice cultivars with distinct agronomic characteristics and genetic backgrounds. CNT is a high-yielding, non-photoperiod-sensitive cultivar, KDML105 is Thailand’s premium aromatic rice cultivar, and RD51 is an aromatic rice cultivar developed from KDML105 with improved agronomic performance. However, comparative information regarding silicon accumulation, phytolith formation, and associated proteomic profiles among these cultivars remains limited. In particular, the molecular mechanisms and biological processes associated with cultivar-dependent variation in silicon uptake, translocation, and phytolith formation at the flowering stage remain poorly understood. Therefore, the objectives of this study were to (i) compare silicon concentration and phytolith accumulation among CNT, KDML105, and RD51 rice cultivars at the flowering stage, (ii) characterize cultivar-specific protein profiles in root and shoot tissues using LC-MS/MS-based proteomic analysis, and (iii) identify biological processes and candidate proteins potentially associated with silicon utilization and phytolith formation. By integrating physiological and proteomic analyses, this study aimed to identify cultivar-dependent mechanisms underlying silicon accumulation and phytolith formation and to evaluate the potential of different rice cultivars for enhanced silicon and phytolith production. The findings provide new insights into the molecular basis of silicon utilization in rice and may support future breeding efforts aimed at improving silicon-use efficiency, stress resilience, and phytolith production.

## 2. Results

### 2.1. Silicon Concentration and Phytolith Content in Rice Cultivars at the Flowering Stage

**Figure 1:**
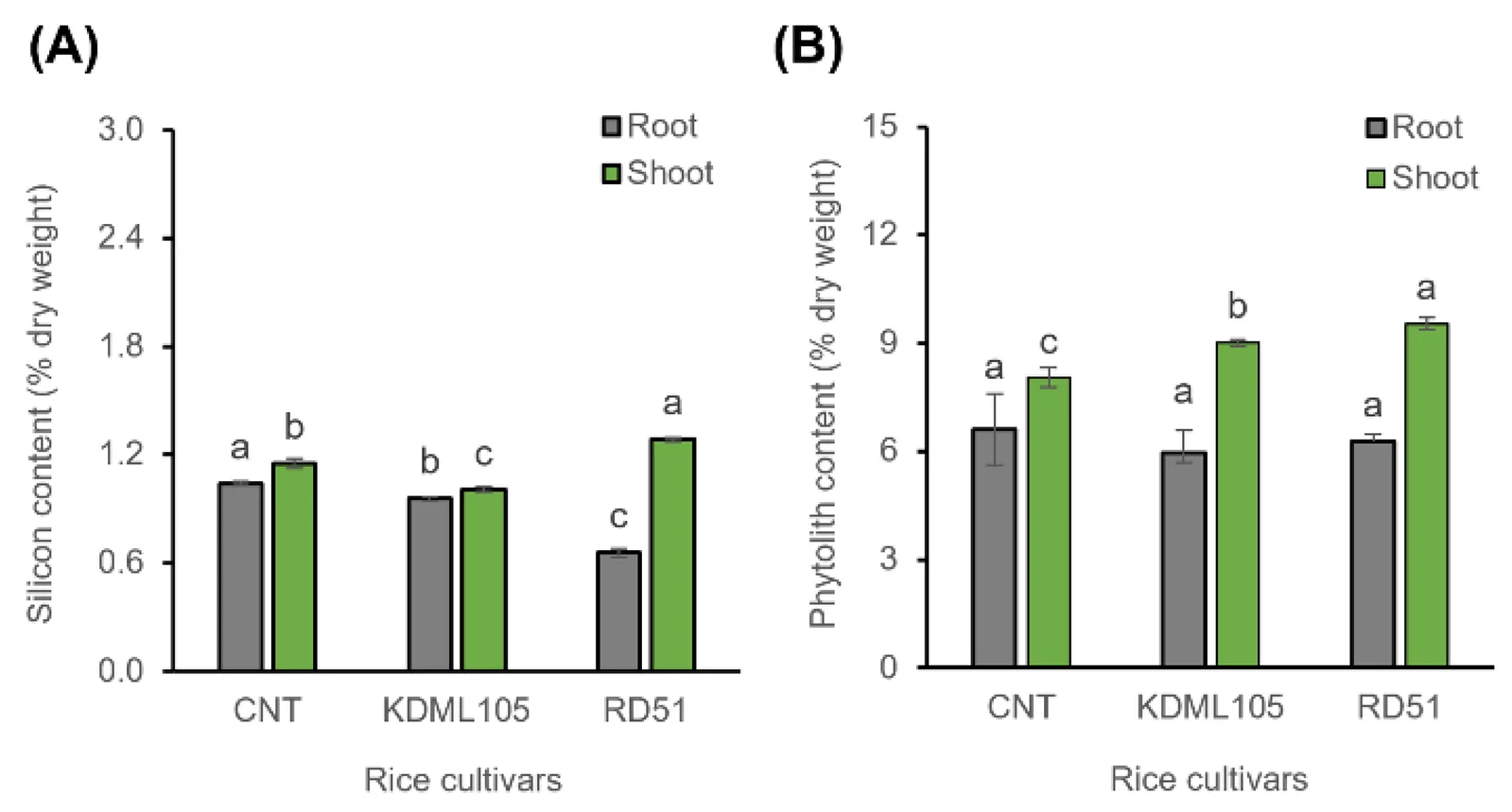
Silicon content (**A**) and phytolith content (**B**) in roots and shoots of the rice cultivars, CNT, KDML105, and RD51, at the flowering stage. Data are presented as mean ± SD (*n* = 3). Different lowercase letters indicate significant differences among cultivars within the same tissue (roots or shoots) according to one-way ANOVA followed by Tukey’s multiple comparison test (*p* < 0.05).

Silicon content differed significantly among rice cultivars in both roots and shoots (Figure 1A). In roots, CNT exhibited the highest silicon content (0.891 ± 0.009% DW), followed by KDML105 (0.820 ± 0.009% DW) and RD51 (0.562 ± 0.021% DW). One-way ANOVA revealed significant differences among cultivars (*F*(2,6) = 467.32, *p* = 2.60 × 10^−7^). Tukey’s multiple comparison test further indicated significant differences among all cultivar pairs, with CNT > KDML105 > RD51. In shoots, RD51 showed the highest silicon content (1.100 ± 0.010% DW), followed by CNT (0.985 ± 0.022% DW) and KDML105 (0.861 ± 0.013% DW). Cultivar significantly affected shoot silicon content (*F*(2,6) = 173.32, *p* = 4.93 × 10^−6^). Tukey’s HSD test demonstrated significant differences among all pairwise comparisons (*p* < 0.001), with RD51 > CNT > KDML105. Phytolith content in roots ranged from 6.0 ± 0.20% DW to 7.0 ± 0.98% DW and did not differ significantly among cultivars (*p* > 0.05; Figure 1B). In contrast, shoot phytolith content differed significantly among cultivars, with RD51 exhibiting the highest value (10.0 ± 0.17% DW), followed by KDML105 (9.0 ± 0.10% DW) and CNT (8.0 ± 0.27% DW).

### 2.2. Protein Clustering

**Figure 2:**
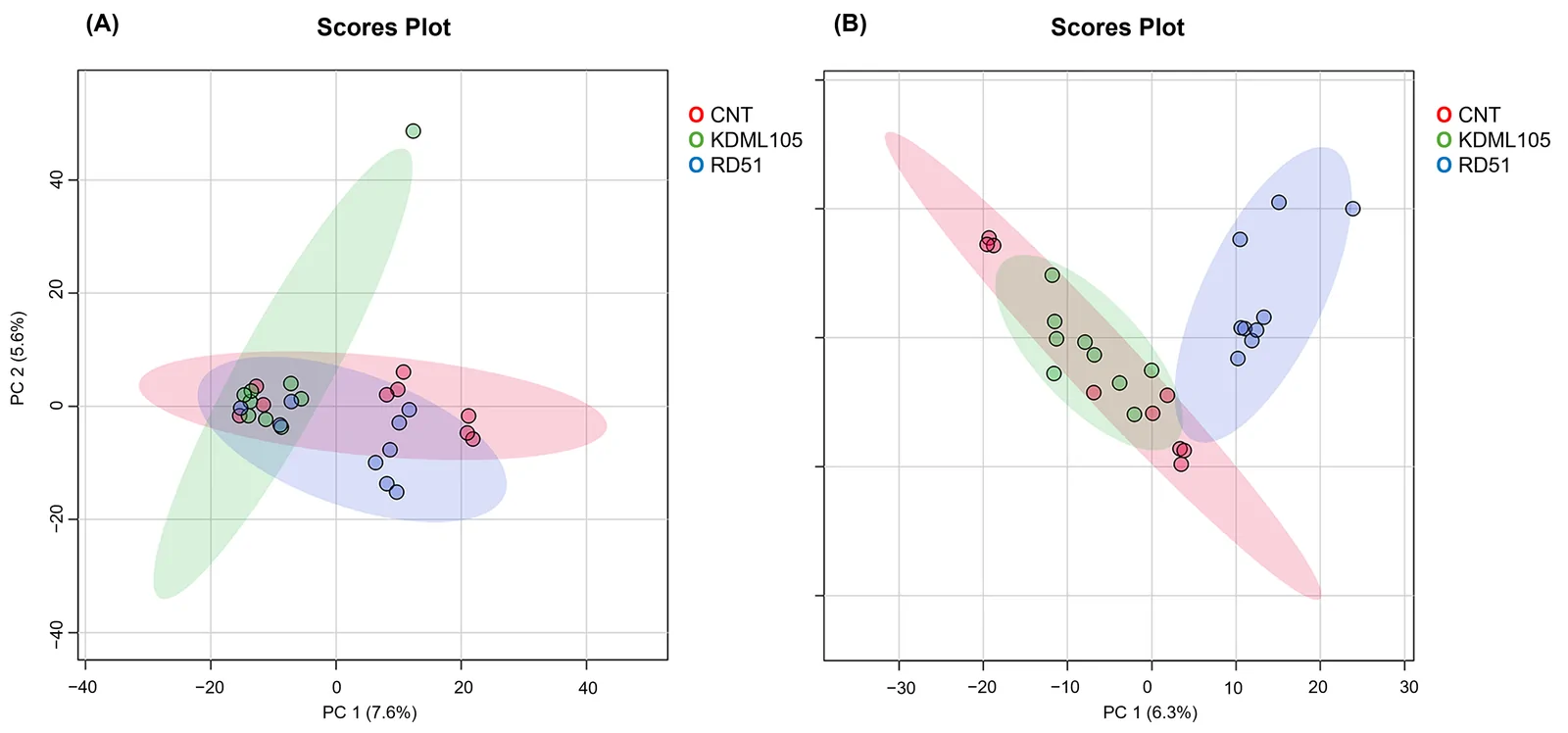
Principal component analysis (PCA) score plots of protein abundance profiles in roots (**A**) and shoots (**B**) of the rice cultivars CNT, KDML105, and RD51. Each point represents an individual LC-MS/MS analysis generated from three biological replicates analyzed in technical triplicate. Shaded ellipses indicate the 95% confidence regions for each cultivar.

A total of 4086 and 4115 protein groups were identified in roots and shoots, respectively (Supplementary Table S1). Principal component analysis (PCA) revealed cultivar-dependent differences in protein abundance profiles in both roots and shoots (Figure 2A,B). In roots, substantial overlap was observed between CNT and KDML105, whereas RD51 showed partial separation from the other cultivars along PC1 (Figure 2A). PC1 and PC2 explained 7.6% and 6.5% of the total variance, respectively. PERMANOVA confirmed significant differences among cultivars (F = 3.757, R^2^ = 0.220, *p* = 0.011). Pairwise comparisons indicated no significant difference between CNT and KDML105 (adjusted *p* = 0.713), whereas RD51 differed significantly from both CNT (adjusted *p* = 0.0015, R^2^ = 0.382) and KDML105 (adjusted *p* = 0.0015, R^2^ = 0.633).

In shoots, a clearer separation among cultivars was observed, with RD51 forming a distinct cluster relative to CNT and KDML105 (Figure 2B). PC1 and PC2 explained 6.3% and 4.6% of the total variance, respectively. PERMANOVA further revealed significant differences among cultivars (F = 10.634, R^2^ = 0.486, *p* = 0.001). Pairwise comparisons showed significant differences between CNT and KDML105 (adjusted *p* = 0.0165, R^2^ = 0.361), CNT and RD51 (adjusted *p* = 0.0015, R^2^ = 0.708), and KDML105 and RD51 (adjusted *p* = 0.0015, R^2^ = 0.633). The largest pairwise effect size was observed between CNT and RD51 (*R^2^* = 0.708), indicating the greatest divergence in shoot protein abundance profiles among the cultivar comparisons.

### 2.3. Differentially Abundant Proteins (DAPs) in Roots and Shoots

**Figure 3:**
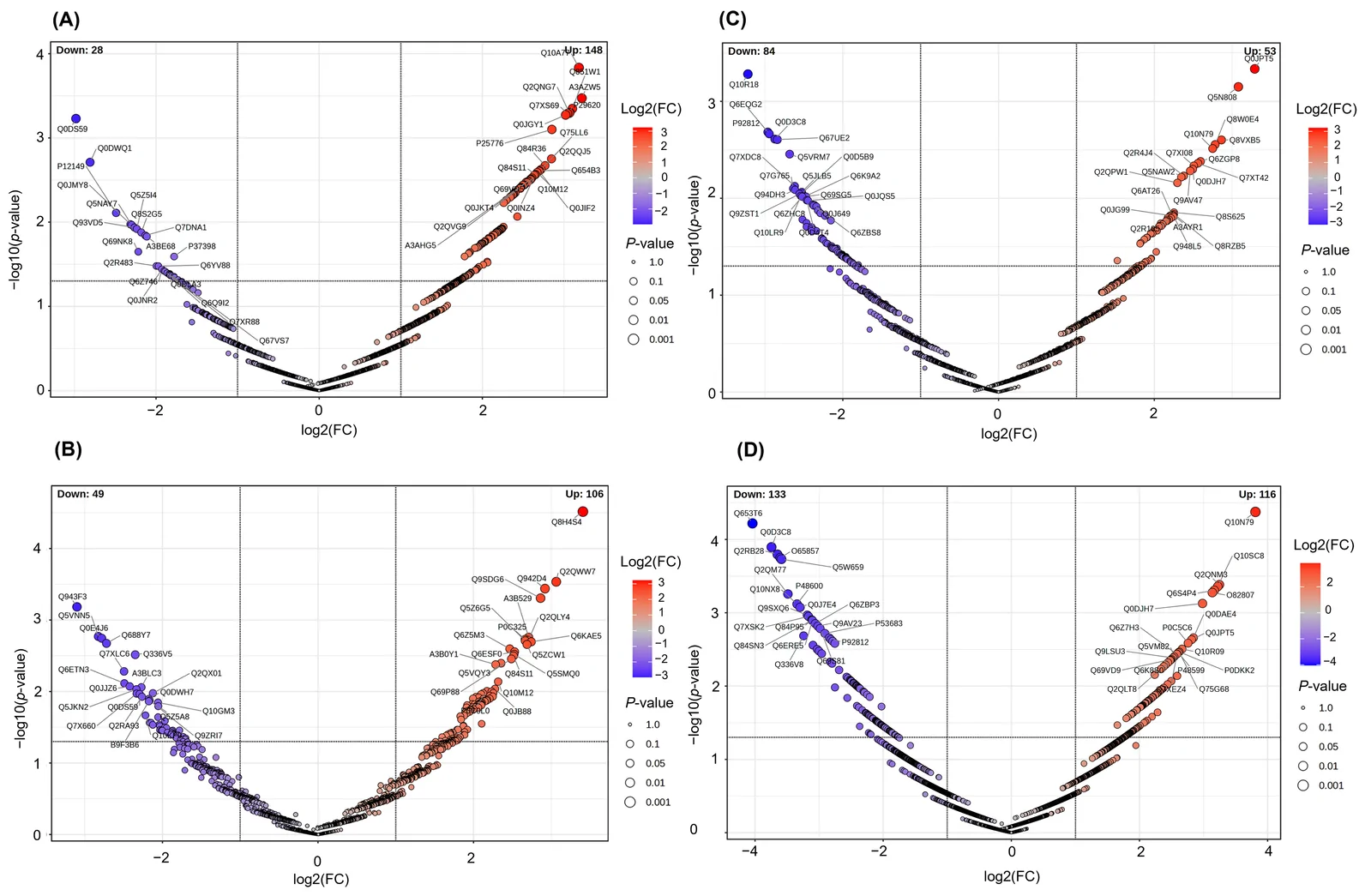
Volcano plots of differentially abundant proteins (DAPs) in CNT vs. KDML105 roots (**A**), CNT vs. RD51 roots (**B**), CNT vs. KDML105 shoots (**C**), and CNT vs. RD51 shoots (**D**). Each point represents an individual protein. Red and blue dots indicate significantly increased (log2FC > 1, *p* < 0.05) and decreased (log2FC < −1, *p* < 0.05) proteins, respectively. Numbers of up- and down-regulated proteins were 148 and 28 (**A**), 106 and 49 (**B**), 53 and 84 (**C**), and 116 and 133 (**D**), respectively. Labeled proteins represent selected DAPs with high fold changes and statistical significance and are identified by their UniProt accession numbers. DAPs were identified using a limma-based moderated *t*-test (*p* < 0.05; fold change > 2). The complete list of DAPs and their annotations is provided in Supplementary Table S2.

Volcano plot analysis revealed extensive cultivar-dependent changes in protein abundance profiles (Figure 3A–D, Supplementary Table S2). In roots, the comparison between CNT and KDML105 identified 176 DAPs, comprising 148 up-regulated and 28 down-regulated proteins (Figure 3A). The most abundant up-regulated proteins were sucrose-phosphatase 3 (A3AZW5; FC = 9.32, log2FC = 3.22, −log10(*p*) = 3.47), protein TIC62 chloroplastic (Q10A77; FC = 9.08, log2FC = 3.18, −log10(*p*) = 3.83), SEC1 family transport protein SLY1 (Q851W1; FC = 8.59, log2FC = 3.10, −log10(*p*) = 3.35), and cyclin-dependent kinase D-1 (P29620; FC = 8.40, log2FC = 3.07, −log10(*p*) = 3.30). In contrast, 155 DAPs were identified between CNT and RD51 roots, including 106 up-regulated and 49 down-regulated proteins (Figure 3B). The most highly accumulated proteins were NAC domain-containing protein 10 (Q8H4S4; FC = 10.60, log2FC = 3.41, −log10(*p*) = 4.52), phosphate transporter 4;7 (Q2QWW7; FC = 8.36, log2FC = 3.06, −log10(*p*) = 3.53), BURP domain-containing protein 3 (Q942D4; FC = 7.56, log2FC = 2.92, −log10(*p*) = 3.44), and 60S ribosomal protein L30 (Q9SDG6; FC = 7.27, log2FC = 2.86, −log10(*p*) = 3.31).

In shoots, 137 DAPs were identified between CNT and KDML105, including 53 up-regulated and 84 down-regulated proteins (Figure 3C). The most abundant up-regulated proteins were aquaporin NIP1-2 (Q0JPT5; FC = 9.78, log2FC = 3.29, −log10(*p*) = 3.34), signal peptide peptidase-like 3 (Q5N808; FC = 8.46, log2FC = 3.08, −log10(*p*) = 3.15), potassium transporter 8 (Q8VXB5; FC = 7.27, log2FC = 2.86, −log10(*p*) = 2.60), and serine acetyltransferase 1 (Q8W0E4; FC = 6.87, log2FC = 2.78, −log10(*p*) = 2.55). The largest proteomic divergence was observed between CNT and RD51 shoots, where 249 DAPs were identified, including 116 up-regulated and 133 down-regulated proteins (Figure 3D). Up-regulated proteins exhibited fold changes ranging from 2.98- to 14.02-fold (log2FC = 1.57–3.81), with −log10(*p*) values reaching 4.38. The most highly accumulated proteins were arginine biosynthesis bifunctional protein ArgJ0 (Q10N79; FC = 14.02, log2FC = 3.81, −log10(*p*) = 4.38), RNA pseudouridine synthase 1 (Q2QNM3; FC = 9.49, log2FC = 3.25, −log10(*p*) = 3.38), CBL-interacting protein kinase 9 (Q10SC8; FC = 9.33, log2FC = 3.22, −log10(*p*) = 3.36), transcription factor RF2b (Q6S4P4; FC = 8.99, log2FC = 3.17, −log10(*p*) = 3.31), and alternative oxidase 1A (O82807; FC = 8.80, log2FC = 3.14, −log10(*p*) = 3.28). Overall, the greatest proteomic variation was observed in the CNT versus RD51 shoot comparison.

### 2.4. Gene Ontology Enrichment Analysis of Up-Regulated DAPs

**Figure 4:**
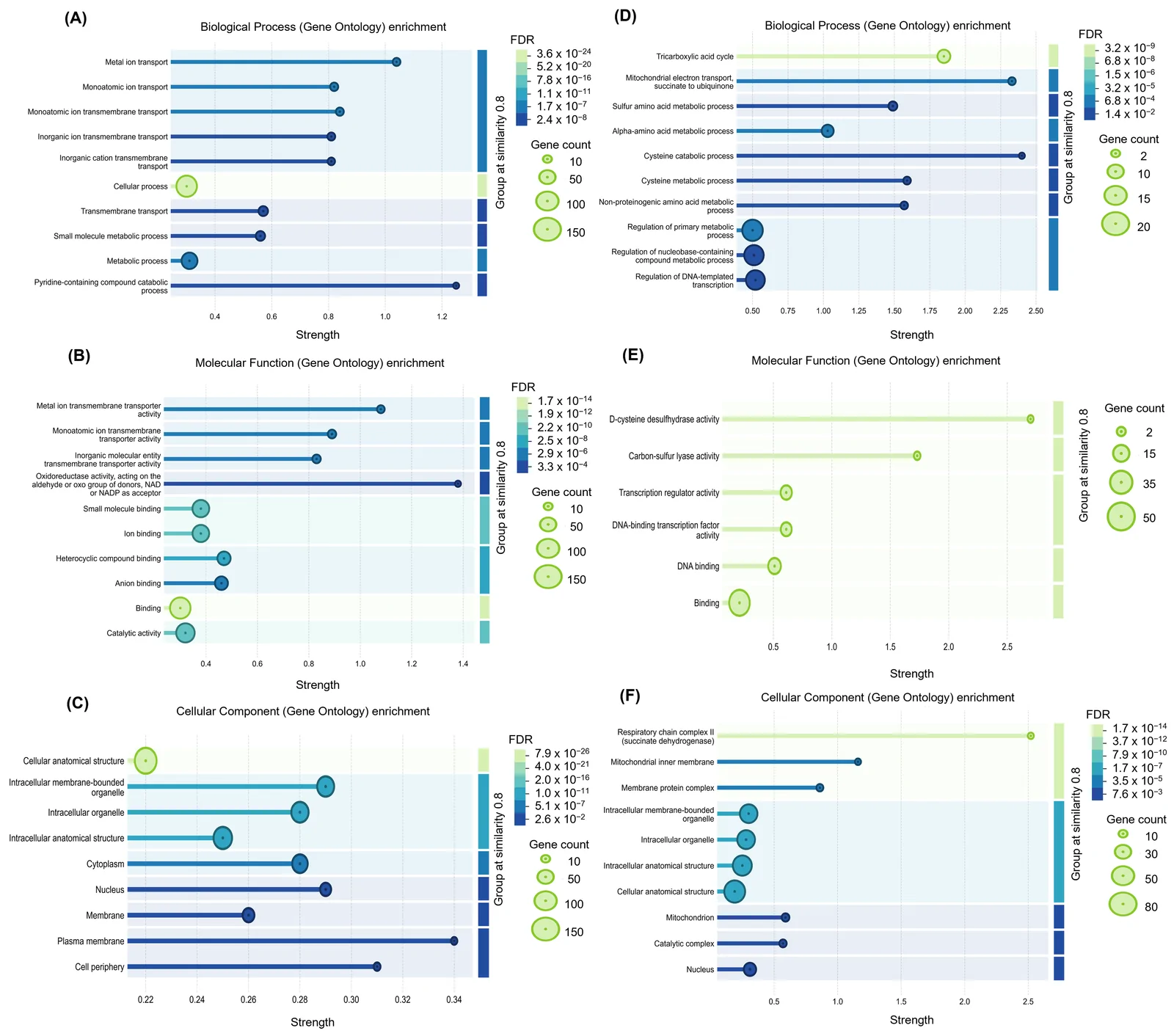
Gene Ontology (GO) enrichment analysis of up-regulated differentially abundant proteins (DAPs) identified in KDML105 roots and shoots. Significantly enriched GO terms were classified into Biological Process (BP), Molecular Function (MF), and Cellular Component (CC). Panels (**A**–**C**) show enriched GO terms in roots, whereas panels (**D**–**F**) show enriched GO terms in shoots. Bubble size represents the number of proteins associated with each GO term, and color indicates the false discovery rate (FDR). The x-axis represents enrichment strength [log10(observed/expected)].

**Figure 5:**
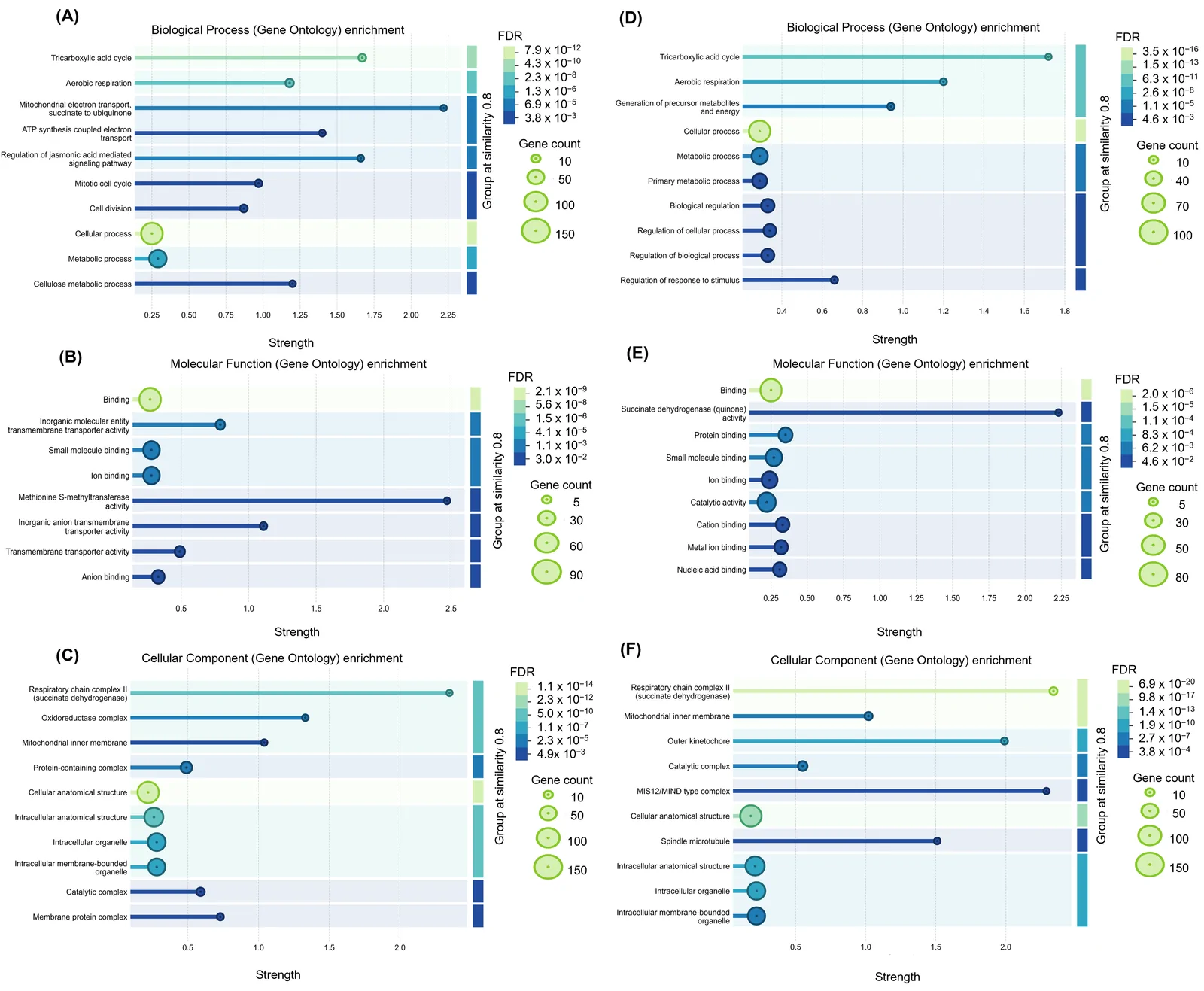
Gene Ontology (GO) enrichment analysis of up-regulated differentially abundant proteins (DAPs) identified in RD51 roots and shoots. Significantly enriched GO terms were classified into Biological Process (BP), Molecular Function (MF), and Cellular Component (CC). Panels (**A**–**C**) show enriched GO terms in roots, whereas panels (**D**–**F**) show enriched GO terms in shoots. Bubble size represents the number of proteins associated with each GO term, and color indicates the false discovery rate (FDR). The x-axis represents enrichment strength [log10(observed/expected)].

Gene Ontology (GO) enrichment analysis of up-regulated DAPs revealed distinct functional enrichment patterns in KDML105 and RD51 roots and shoots (Figure 4 and Figure 5). In KDML105 roots, enriched biological processes were mainly associated with metal ion transport, vesicle-mediated transport, transmembrane transport, and inorganic anion transmembrane transport (Figure 4A). Enriched molecular functions included transporter activity, transmembrane transporter activity, inorganic anion transmembrane transporter activity, and ion binding (Figure 4B). Enriched cellular components were primarily associated with the cell periphery, plasma membrane, and cell wall-related structures (Figure 4C). In KDML105 shoots, enriched biological processes were predominantly related to sulfur amino acid metabolism, sulfur amino acid biosynthetic processes, cysteine metabolic processes, cysteine biosynthetic processes, and regulation of primary metabolic processes (Figure 4D). Enriched molecular functions included L-cysteine desulfurase activity, cysteine lyase activity, transcription regulator activity, and DNA-binding transcription factor activity (Figure 4E). Enriched cellular components were associated with respiratory chain complexes, mitochondrial membrane protein complexes, and intracellular membrane-bounded organelles (Figure 4F). In contrast, RD51 exhibited stronger enrichment of energy metabolism- and respiration-related functions (Figure 5A–F). In roots, enriched biological processes included the tricarboxylic acid cycle, aerobic respiration, mitochondrial electron transport coupled to ATP synthesis, ATP synthesis-coupled electron transport, and regulation of jasmonic acid-mediated signaling pathways (Figure 5A). Enriched molecular functions included methionine adenosyltransferase activity, transporter activity, amino acid binding, and ion binding (Figure 5B), whereas enriched cellular components were primarily associated with respiratory chain complexes, mitochondrial inner membranes, and catalytic complexes (Figure 5C). Similarly, in shoots, enriched biological processes were associated with the tricarboxylic acid cycle, aerobic respiration, generation of precursor metabolites and energy, primary metabolic processes, and regulation of cellular processes (Figure 5D). Enriched molecular functions included succinate dehydrogenase activity, catalytic activity, metal ion binding, and nucleic acid binding (Figure 5E). Enriched cellular components were dominated by respiratory chain complexes, mitochondrial inner membranes, membrane protein complexes, and catalytic complexes (Figure 5F). Overall, KDML105 was characterized by enrichment of transport- and sulfur metabolism-related functions, whereas RD51 displayed pronounced enrichment of mitochondrial respiration and energy production pathways. The complete GO enrichment results are provided in Supplementary Table S3.

### 2.5. Protein–Protein Interaction (PPI) Network Analysis of Up-Regulated DAPs and Silicon Transporter Proteins

**Figure 6:**
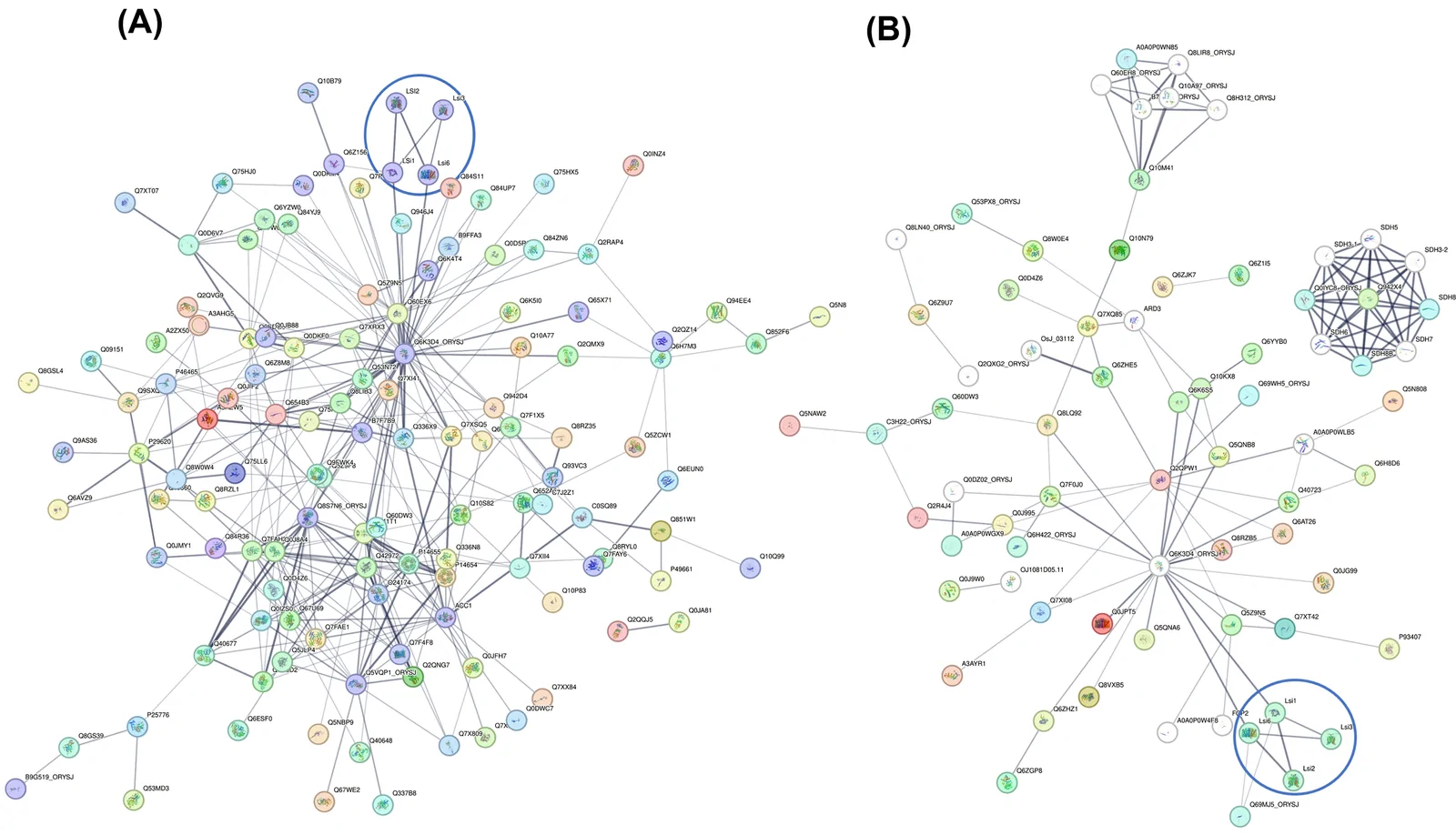
Protein–protein interaction (PPI) networks of up-regulated proteins identified in KDML105 roots (**A**) and shoots (**B**). Networks were generated using STRING v12.5 with an interaction score threshold of ≥0.400. Nodes represent proteins, and edges represent predicted functional associations. Silicon transporter proteins (Lsi1, Lsi2, Lsi3, and Lsi6) are highlighted with blue circles.

**Figure 7:**
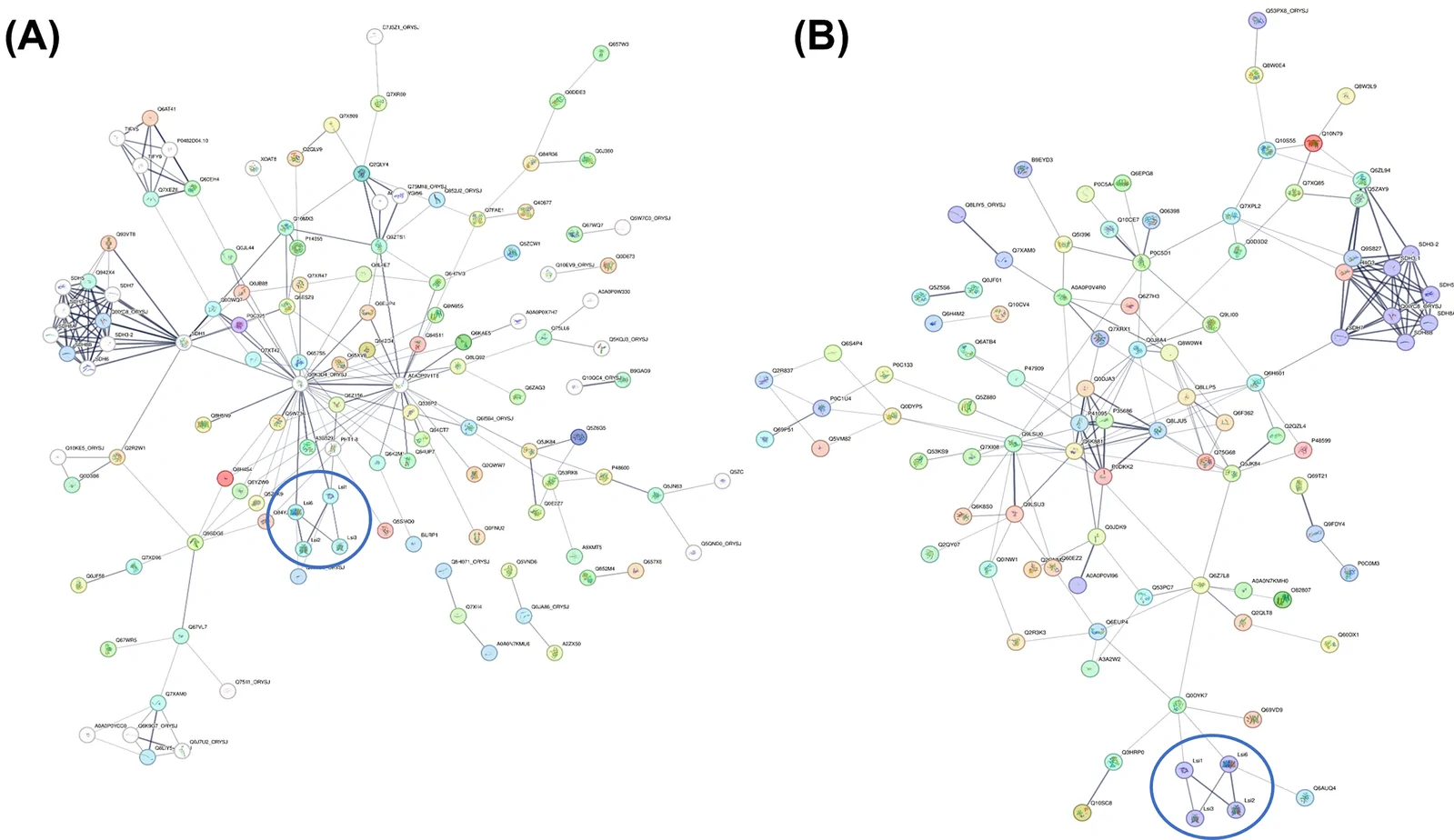
Protein–protein interaction (PPI) networks of up-regulated proteins identified in RD51 roots (**A**) and shoots (**B**). Networks were generated using STRING v12.5 with an interaction score threshold of ≥0.400. Nodes represent proteins, and edges represent predicted functional associations. Silicon transporter proteins (Lsi1, Lsi2, Lsi3, and Lsi6) are highlighted with blue circles.

To further elucidate the functional relationships between silicon transport and proteomic responses, protein–protein interaction (PPI) networks were constructed using STRING analysis by integrating the silicon transporter proteins Lsi1, Lsi2, Lsi3, and Lsi6 with the differentially abundant proteins (DAPs) identified in root and shoot tissues of the rice cultivars KDML105 and RD51 (Figure 6 and Figure 7). In KDML105 roots, the resulting network comprised 157 nodes and 318 edges, significantly exceeding the 258 interactions expected by chance (PPI enrichment *p* = 0.000167) (Figure 6A; Supplementary Table S4). The network was centered on the highly connected hub protein Q6K3D4 and consisted of several interconnected subnetworks. A distinct silicon transporter module comprising Lsi1, Lsi2, Lsi3, and Lsi6 was identified and connected to the core network through Q6K3D4 and PHR2 (Q6Z156). In addition, proteins involved in transport, signal transduction, and metabolic processes were integrated into the network, forming extensive interactions among silicon transport-associated proteins and other regulatory components.

In KDML105 shoots, the STRING analysis generated a PPI network consisting of 87 nodes and 127 edges, significantly exceeding the 61 interactions expected by chance (PPI enrichment *p* = 1.36 × 10^−13^) (Figure 6B; Supplementary Table S5). The network was centered on the highly connected hub protein Q6K3D4 and comprised several interconnected subnetworks. A distinct silicon transporter module consisting of Lsi1, Lsi2, Lsi3, and Lsi6 was directly interconnected and linked to the core network through Q6K3D4. In addition, a highly interconnected succinate dehydrogenase (SDH) cluster comprising SDH3-1, SDH3-2, SDH4, SDH5, SDH6, SDH7, SDH8A, and SDH8B was identified. The network also contained proteins associated with transcriptional regulation, stress responses, ion transport, and carbohydrate metabolism, including SPL7, HSFB4D, HAK8, SPS4, and TPP4, indicating extensive functional interactions among transport, regulatory, and metabolic components in KDML105 shoots.

In RD51 roots, the STRING-derived PPI network comprised 149 nodes and 221 edges, significantly exceeding the 157 interactions expected by chance (PPI enrichment *p* = 7.07 × 10^−7^), with an average node degree of 2.97 and an average local clustering coefficient of 0.506 (Figure 7A; Supplementary Table S6). The network was centered on the highly connected hub protein Q6K3D4 (degree = 30), followed by SDH1 (degree = 17), and SDH3-1 and SDH3-2 (degree = 10). A distinct silicon transporter module comprising Lsi1, Lsi2, Lsi3, and Lsi6 was connected to the core network through Q6K3D4 and PHR2. The network further incorporated proteins associated with nutrient transport, signal transduction, transcriptional regulation, hormone signaling, and cell wall metabolism, indicating extensive interactions among silicon transport-associated proteins and multiple regulatory pathways in RD51 roots. In addition, a prominent succinate dehydrogenase (SDH) subnetwork comprising SDH1, SDH3-1, SDH3-2, SDH5, SDH6, SDH7, SDH8A, and SDH8B was evident within the network.

In RD51 shoots, the STRING-derived PPI network comprised 130 nodes and 187 edges, exceeding the 144 interactions expected by chance (PPI enrichment *p* = 0.000335), with an average node degree of 2.88 and an average local clustering coefficient of 0.365 (Figure 7B; Supplementary Table S7). The network was centered on phosphatidylinositol 4-kinase gamma 4 (Q6K881; degree = 18), followed by succinate dehydrogenase iron-sulfur subunit 1 (SDH2-1; degree = 12), succinate dehydrogenase iron-sulfur subunit 2 (SDH2-2; degree = 11), and Q6H4G3 (degree = 11). A distinct silicon transporter module consisting of Lsi1, Lsi2, Lsi3, and Lsi6 was connected to the network through calcium signaling-related proteins, including CBL7 and CIPK9. The network also contained proteins associated with calcium-mediated signaling, transcriptional regulation, hormone signaling, energy metabolism, redox homeostasis, and stress-responsive processes. Furthermore, a highly interconnected SDH subnetwork comprising SDH2-1, SDH2-2, SDH3-1, SDH3-2, SDH5, SDH7, SDH8A, and SDH8B was evident within the network. Overall, the RD51 shoot network revealed extensive interactions among silicon transport, calcium-mediated signaling, respiratory metabolism, and stress-associated pathways.

## 3. Discussion

### 3.1. Cultivar-Dependent Silicon Accumulation and Phytolith Formation

The present study revealed significant cultivar-dependent variation in silicon accumulation and phytolith formation in rice at the flowering stage (Figure 1). Although CNT accumulated the highest silicon concentration in roots, RD51 exhibited significantly greater silicon accumulation and phytolith content in shoots, whereas KDML105 showed intermediate values. These contrasting distribution patterns indicate that genetic variation influences not only silicon uptake but also the efficiency of silicon partitioning between root and shoot tissues. The relatively low silicon concentration in RD51 roots coupled with its high shoot silicon concentration suggests enhanced root-to-shoot silicon translocation compared with CNT and KDML105. Silicon uptake and distribution in rice are regulated by the coordinated activities of the transporters Lsi1, Lsi2, Lsi3, and Lsi6 [6,16,17,18]. Lsi1 facilitates silicic acid influx into root cells, whereas Lsi2 mediates silicon efflux toward the stele and subsequent xylem loading [6,16]. Following uptake, silicon is redistributed to aerial tissues through the actions of Lsi3 and Lsi6, where silica deposition predominantly occurs [8,17]. In this context, the lower silicon concentration observed in RD51 roots together with its enhanced shoot accumulation (Figure 1A) is consistent with more efficient internal silicon transport and allocation. A strong positive association between shoot silicon accumulation and phytolith formation was evident in the present study. RD51 exhibited the highest shoot silicon concentration and phytolith content among the evaluated cultivars (Figure 1A,B). Because phytoliths are produced through the polymerization of soluble silicic acid into amorphous silica, increased silicon availability generally promotes phytolith formation [6,19]. Similar relationships have been reported in rice and other silicon-accumulating grasses, where elevated silicon accumulation enhances silica deposition in leaves, stems, and leaf sheaths [18,19]. Moreover, Luangcharuthorn et al. [20] reported that Thai rice cultivars with high phytolith accumulation displayed increased expression of *OsLsi2* and *OsLsi3*, supporting the importance of efficient silicon transport and redistribution for phytolith production. Despite significant differences in root silicon concentration, root phytolith content remained comparable among cultivars (Figure 1B). This observation indicates that a substantial proportion of silicon retained in roots remains in a mobile form available for translocation to aerial tissues. Such a distribution pattern is consistent with reports demonstrating that silicon deposition occurs predominantly in transpiring tissues, particularly leaves and leaf sheaths, which represent the primary sites of phytolith formation in rice [9,21]. Consequently, cultivar-dependent variation was considerably more pronounced in shoot phytolith accumulation than in root phytolith accumulation. The superior shoot silicon accumulation and phytolith production observed in RD51 may have important physiological consequences. Silicon deposition improves mechanical strength, plant architecture, and lodging resistance while enhancing tolerance to biotic and abiotic stresses [22,23]. Therefore, the greater phytolith production observed in RD51 reflects not only enhanced silicon accumulation but also more efficient silicon utilization in aboveground tissues. Collectively, these findings indicate that phytolith formation is more closely associated with silicon allocation efficiency than with root silicon accumulation alone and identify root-to-shoot silicon translocation as a major factor underlying cultivar-dependent differences in phytolith production. The superior silicon accumulation and phytolith production observed in RD51 also provide a physiological basis for the cultivar-specific proteomic patterns discussed below.

### 3.2. Proteomic Analysis Associated with Silicon Accumulation and Phytolith Formation

Comparative proteomic analysis revealed substantial cultivar-dependent variation in protein abundance associated with differences in silicon accumulation and phytolith formation. Principal component analysis and PERMANOVA demonstrated clear differentiation among cultivars, particularly in shoot tissues, where the greatest proteomic divergence was observed between CNT and RD51 (R^2^ = 0.708) (Figure 2B). The pronounced separation of RD51 from the other cultivars corresponded closely with its superior shoot silicon accumulation and phytolith production (Figure 1), indicating that these physiological traits are accompanied by extensive proteome remodeling. Functional enrichment analysis revealed distinct biological characteristics among cultivars. KDML105 was primarily enriched in proteins associated with transport processes, transmembrane trafficking, vesicle-mediated transport, and sulfur amino acid metabolism (Figure 4), whereas RD51 showed pronounced enrichment of proteins involved in the tricarboxylic acid cycle, aerobic respiration, mitochondrial electron transport, and ATP synthesis in both roots and shoots (Figure 5). Mitochondrial respiration is central to ATP production and maintenance of electrochemical gradients required for active transport processes [24,25]. The enrichment of respiration-related pathways in RD51 therefore suggests an enhanced capacity for energy generation that may support efficient silicon redistribution from roots to shoots. This interpretation is supported by the physiological observations. Although RD51 accumulated less silicon in roots than CNT, it exhibited the highest silicon concentration and phytolith content in shoots (Figure 1). Silicon translocation involves coordinated membrane transport, xylem loading, intracellular trafficking, and tissue-specific redistribution [16,17], all of which require substantial metabolic energy. Consequently, the enrichment of mitochondrial metabolic pathways observed in RD51 may provide the energetic basis for enhanced silicon allocation and silica deposition in aerial tissues. Several highly abundant proteins identified in RD51 shoots further support the involvement of signaling and metabolic regulation in silicon utilization. Among these, CIPK9 is a component of the CBL-CIPK signaling network, which regulates nutrient homeostasis, ion transport, and adaptive stress responses [26,27,28]. In addition, AOX1A, a key component of the mitochondrial alternative respiratory pathway, contributes to respiratory flexibility and maintenance of cellular redox balance under fluctuating metabolic conditions [29,30]. Increased abundance of these proteins suggests coordinated regulation of nutrient signaling, respiratory metabolism, and stress-response pathways in RD51. Protein–protein interaction analysis provided additional insight into the molecular networks associated with silicon accumulation. Integration of the silicon transporters Lsi1, Lsi2, Lsi3, and Lsi6 with differentially abundant proteins generated highly connected interaction networks in both KDML105 and RD51 (Figure 6 and Figure 7). These networks incorporated proteins involved in nutrient transport, signal transduction, transcriptional regulation, and energy metabolism, indicating that silicon transport operates within a broader regulatory framework rather than as an isolated process. A prominent feature of the RD51 networks was the presence of extensive succinate dehydrogenase (SDH) subnetworks in both roots and shoots (Figure 7). SDH functions at the interface of the tricarboxylic acid cycle and the mitochondrial electron transport chain and therefore plays a critical role in energy production [30]. The enrichment of SDH-related proteins is consistent with the GO enrichment results (Figure 5) and further supports the importance of respiratory metabolism in facilitating silicon allocation within this cultivar. The RD51 shoot network additionally revealed associations between silicon transporters and calcium-signaling proteins, including CBL7 and CIPK9 (Figure 7B). Calcium signaling represents a central regulatory system controlling nutrient transport, environmental perception, and adaptive responses in plants [26,27]. The incorporation of calcium-signaling components into the silicon transporter-associated network suggests that silicon homeostasis is integrated with signaling pathways that regulate nutrient redistribution and cellular adaptation during reproductive development.

Overall, the proteomic and network analyses indicate that cultivar-dependent variation in silicon accumulation and phytolith formation is associated with coordinated changes in transport-related functions, mitochondrial respiration, calcium-mediated signaling, and cellular homeostasis. The superior silicon accumulation and phytolith production observed in RD51 were accompanied by enrichment of energy-generating pathways and highly interconnected regulatory networks, suggesting enhanced silicon allocation efficiency. These findings provide new insight into the molecular basis of silicon utilization in rice and identify candidate proteins and biological processes potentially involved in silicon translocation and phytolith formation. Future studies integrating quantitative expression analysis, targeted proteomics, and functional characterization of transporter-associated proteins will further clarify the molecular mechanisms regulating silicon homeostasis and phytolith production in rice.

## 4. Materials and Methods

### 4.1. Plant Materials, Growing Conditions, Experimental Design and Sample Collection

Seeds of three rice (*Oryza sativa* L. ssp. *indica*) cultivars, namely Chai Nat 1 (CNT; reference cultivar), Khao Dawk Mali 105 (KDML105; jasmine rice), and RD51 (flash-flood-tolerant rice) (Table 1), were obtained from the Pathum Thani Rice Research Center, Rice Department, Ministry of Agriculture and Cooperatives, Thailand. Rice seeds were soaked in distilled water for 48 h and subsequently germinated on sterile sand for 2 weeks. The seedlings were then transplanted into cement pots (3.4 m × 1.5 m × 0.41 m) containing air-dried soil to a depth of 0.30 m and maintained under regular irrigation until harvest. Basal fertilizer (16-20-0 ammonium phosphate) was applied at a rate of 20 kg rai^−1^, 14 days before transplanting. Plants were cultivated in a greenhouse under a photosynthetic photon flux density of 825 ± 50 μmol m^−2^ s^−1^, with day and night temperatures maintained at 32 ± 2 °C and 28 ± 2 °C, respectively. The experiment was arranged in a randomized complete block design (RCBD) consisting of three blocks. Each block contained all three rice cultivars (CNT, KDML105, and RD51), and cultivar positions were randomly assigned within each block to minimize the effects of environmental and soil heterogeneity. The experiment included three biological replicates for silicon concentration and phytolith analyses (*n* = 3). Plants were harvested at the flowering stage (60 days after planting). Harvested plants were thoroughly washed with tap water and separated into roots and shoots (leaf blades, sheaths, and stems). Samples were dried in a hot-air oven at 60 °C for 48 h and subsequently used to determine silicon concentration and phytolith content. For proteomic analysis, fresh root and shoot tissues were cut into small pieces, immediately frozen in liquid nitrogen, and stored at −80 °C until analysis. Proteomic analyses were performed using three independent biological replicates (*n* = 3), with each replicate processed and analyzed separately without pooling.

### 4.2. Silicon Concentration Analysis

Silicon concentration was determined according to the method of Dai et al. [31] with minor modifications. Dried plant material was ground into a fine powder, and 0.05 g of each sample was extracted with 600 μL of 50% NaOH and autoclaved at 121 °C for 20 min. Extracts were diluted to 25 mL with distilled water and filtered through Whatman No. 1 filter paper. Aliquots (200 μL) of the filtrate were transferred to a 50 mL volumetric flask, followed by the addition of 6 mL of 20% acetic acid and 2 mL of ammonium molybdate solution (pH 7.0). After incubation for 5 min at room temperature, 1 mL of 20% tartaric acid and 200 μL of reducing solution were added. The final reaction volume was adjusted to 10 mL with 20% acetic acid. Absorbance was measured at 650 nm after 30 min using a Multiskan GO microplate spectrophotometer (Thermo Fisher Scientific, Waltham, MA, USA).

Phytolith content in root and shoot tissues was determined using the dry ash method described by Parr et al. [32]. Dried tissues were cut into small pieces, and approximately 4 g (dry weight) was ground and transferred to crucibles. Samples were ashed in a muffle furnace at 500 °C for 8 h. The resulting ash was treated with 10% HCl at 70 °C for 20 min and centrifuged at 12,000× *g* for 5 min. The residue was washed with distilled water and subsequently treated with 15% H_2_O_2_ at 70 °C until the reaction ceased. After repeated washing and centrifugation, the final residue was suspended in 1 mL of absolute ethanol and allowed to dry overnight. The extracted phytoliths were oven-dried at 70 °C for 24 h, mounted on glass slides, and examined under a microscope (Jiangnan XP-213, Nanjing, China) equipped with a polarizing filter and a 5.0 MP color CCD camera at 400× magnification to confirm the absence of residual organic material. Phytolith content was expressed as a percentage of plant dry weight (% DW). Silicon concentration and phytolith content are presented as mean ± standard deviation (SD). Statistical differences among rice cultivars were evaluated by one-way analysis of variance (ANOVA) followed by Tukey’s multiple comparison test. Differences were considered significant at *p* < 0.05. Statistical analyses were performed using Minitab version 18.0 (Minitab Inc., Coventry, UK).

### 4.3. Protein Extraction and In-Solution Digestion

Samples were washed with sterile distilled water and ground into a fine powder in liquid nitrogen. Protein extraction was performed from 0.5 g of powdered tissue following Saekampang et al. [33]. Briefly, proteins were extracted using 0.5% (*w*/*v*) SDS, precipitated with 0.15% sodium deoxycholate and 72% trichloroacetic acid, washed with cold acetone, and reconstituted in 0.5% SDS. Protein concentration was determined using the Lowry assay with bovine serum albumin (BSA) as the standard. Subsequently, 5 µg of total protein from each sample was digested in solution for shotgun proteomic analysis. Each treatment consisted of three biological replicates, each processed individually and not pooled.

### 4.4. LC-MS/MS Analysis

Three technical LC-MS/MS runs were conducted per replicate. The tryptic peptide samples were performed for injection into an Ultimate 3000 Nano/Capillary LC System (Thermo Fisher Scientific, Loughborough, UK) coupled to a ZenoTOF 7600 mass spectrometer (SCIEX, Framingham, MA, USA). Peptide digests (100 ng) were loaded onto a µ-Precolumn (300 µm i.d. × 5 mm, C18 PepMap 100, 5 μm, 100 Å) and separated on a 75 µm i.d. × 15 cm Acclaim PepMap RSLC C18 column (2 μm, 100 Å, nanoViper, Thermo Scientific, Waltham, MA, USA)) at a flow rate of 0.3 µL min^−1^ over 30 min. Instrument parameters were set according to Ploypetch et al. [34]. The mass spectrometer was operated in positive ion mode with ion source gas 1 at 8 psi, curtain gas at 35 psi, CAD gas at 7 psi, source temperature of 200 °C, and spray voltage of 3.3 kV. Data-dependent acquisition (DDA) mode was employed, selecting the 50 most intense precursor ions above 150 cps for MS/MS analysis. Dynamic exclusion was set to 12 s after two occurrences. MS/MS spectra were acquired over an m/z range of 100–1800 with an accumulation time of 50 ms. The Zeno trap threshold was set to 150,000 cps, and the cycle time was 3.0 s.

Raw LC-MS/MS data were processed using MaxQuant version 2.5.0.0 with the integrated Andromeda search engine. MS/MS spectra were searched against the *Oryza sativa* protein sequence database obtained from UniProtKB (UniProt Consortium; https://www.uniprot.org/, accessed on 5 November 2025). The database contained 61,200 protein entries. Trypsin was selected as the proteolytic enzyme, allowing up to two missed cleavages. The precursor mass tolerance was set to 20 ppm for the first search and 10 ppm for the main search, and the fragment ion mass tolerance was set to 25 ppm. Carbamidomethylation of cysteine was specified as a fixed modification, whereas methionine oxidation and protein N-terminal acetylation were considered variable modifications. Protein and peptide identifications were accepted at a false discovery rate (FDR) of 1% at both the peptide and protein levels. Only proteins identified with at least one unique peptide and a minimum peptide length of seven amino acids were retained for further analysis.

### 4.5. Data Processing, Protein Identification, and Bioinformatic Analysis

The LFQ intensities obtained from MaxQuant were used for quantitative analysis. Data preprocessing, visualization, and statistical evaluation were performed using MetaboAnalyst 6.0 [35]. Proteins detected were filtered using an interquartile range filter (40%) and a low-abundance filter based on median intensity (10%) to remove non-informative variables. Missing values were imputed with one-fifth of the minimum positive value of each protein, assuming that undetected signals fell below the limit of detection. The data were then normalized to the sample median, log2-transformed, and Pareto-scaled. Multivariate analyses, including principal component analysis (PCA) was performed on the processed data. Differentially abundant proteins (DAPs) among rice cultivars were determined using the limma-based moderated *t*-test implemented in MetaboAnalyst 6.0. CNT served as the reference cultivar for all pairwise comparisons (CNT vs. KDML105 and CNT vs. RD51). Protein abundance changes were expressed as fold changes relative to CNT. Proteins meeting the criteria of a raw *p*-value < 0.05 and an absolute log_2_ fold change (|log_2_FC|) ≥ 1 (equivalent to a fold change ≥ 2 or ≤ 0.5) were designated as differentially abundant proteins (DAPs). Proteins with log_2_FC ≥ 1 were considered upregulated, whereas proteins with log_2_FC ≤ −1 were considered downregulated relative to the reference cultivar (CNT). Volcano plots were employed to illustrate both the magnitude of abundance changes and their statistical significance. Gene Ontology (GO) enrichment and protein–protein interaction (PPI) network analyses were performed using STRING v12.5 (https://string-db.org; accessed on 1 October 2026). Significantly upregulated proteins were used as the query set and analyzed against the Gene Ontology Biological Process database. GO terms with a false discovery rate (FDR) ≤ 0.05 were considered significantly enriched. Enriched terms were ranked according to enrichment strength [log_10_(observed/expected)] and visualized as dot plots. To investigate potential functional associations with silicon transport, the rice silicon transporter proteins Lsi1, Lsi2, Lsi3, and Lsi6 were manually incorporated into the STRING analysis because they represent key components of silicon uptake and translocation pathways in rice. Protein–protein interaction networks were subsequently constructed using a minimum required interaction score of 0.400 (medium confidence).

## 5. Conclusions

This study demonstrated significant cultivar-dependent differences in silicon accumulation, phytolith formation, and protein abundance profiles among CNT, KDML105, and RD51 rice cultivars at the flowering stage. CNT accumulated the highest silicon concentration in roots, whereas RD51 exhibited the highest silicon concentration and phytolith content in shoots, suggesting enhanced silicon translocation to aerial tissues. Proteomic analysis identified clear cultivar-dependent differences in protein abundance profiles, with the greatest proteomic divergence occurring between CNT and RD51, particularly in shoots. Functional enrichment analysis indicated that KDML105 was characterized by transport- and sulfur metabolism-related functions, whereas RD51 displayed pronounced enrichment of mitochondrial respiration and energy production pathways. Protein–protein interaction (PPI) network analysis further revealed functional associations between silicon transporters and proteins involved in transport, signaling, and respiratory metabolism. Collectively, these findings suggest that enhanced transport capacity and respiratory metabolism may contribute to efficient silicon translocation and phytolith formation in RD51. The results provide new insights into the proteomic mechanisms underlying silicon utilization in rice and identify potential targets for future studies and breeding programs aimed at improving stress tolerance, productivity, and silicon-use efficiency.

## Figures and Tables

**Table 1:** Characteristics of rice cultivars used in this study.

Common Name	Breeding Name	Parents	Yield (kg ha^−1^)	Dominant Characteristics
Chai Nat 1 (CNT)	Chainat 1	(IR13146-158-1 × IR15314-43-2-3-3) × BKN6995-16-1-1-2	4688	Non-photoperiod-sensitive; high-yielding cultivar; maturity at approximately 130 days after planting.
Khao Dawk Mali 105 (KDML105)	KDML4-2-105	Pure-line selection from a local landrace	2268	Photoperiod-sensitive aromatic rice; characteristic jasmine fragrance; maturity at approximately 120 days after planting.
RD51	RGDU99003-1012-B-2-6-B	KDML105 × IR49830-7-1-2-2	3350	Photoperiod-sensitive aromatic rice; flash-flood tolerant; improved derivative of KDML105; maturity at approximately 95 days after planting.

## Data Availability

The mass spectrometry proteomics data have been deposited in the ProteomeXchange Consortium via the jPOST partner repository under the dataset identifiers PXD085275 (ProteomeXchange) and JPST004980 (jPOST). During the review process, the data can be accessed at https://repository.jpostdb.org/preview/8150127846ac6c954059e9 (accessed on 7 October 2026) using the access key 1244.

## Supplementary Materials

The following supporting information can be downloaded at: https://www.mdpi.com/article/10.3390/ijms27219917/s1.

## Abbreviations

The following abbreviations are used in this manuscript:

ANOVA	Analysis of Variance
BSA	Bovine Serum Albumin
CAD	Collision-Activated Dissociation
CNT	Chai Nat 1
DDA	Data-Dependent Acquisition
FDR	False Discovery Rate
GO	Gene Ontology
KDML105	Khao Dawk Mali 105
LC-MS/MS	Liquid Chromatography–Tandem Mass Spectrometry
LFQ	Label-Free Quantification
Lsi1	Low Silicon 1
Lsi2	Low Silicon 2
Lsi3	Low Silicon 3
Lsi6	Low Silicon 6
NIP	Nodulin 26-Like Intrinsic Protein
PCA	Principal Component Analysis
PERMANOVA	Permutational Multivariate Analysis of Variance
PPI	Protein–Protein Interaction
RCBD	Randomized Complete Block Design
RD51	Rice Department 51
SD	Standard Deviation
SDS	Sodium Dodecyl Sulfate
SDH	Succinate Dehydrogenase
Si	Silicon
STRING	Search Tool for the Retrieval of Interacting Genes/Proteins
UniProtKB	UniProt Knowledgebase
